# Seroprevalence of *Brucella* spp. and Rift Valley fever virus infections in communal pastoral cattle at the wildlife-livestock interface, Zambezi region, Namibia

**DOI:** 10.3389/fvets.2024.1489815

**Published:** 2024-12-12

**Authors:** Oscar Madzingira, Hannah Munzel, Nicky Mowa Simasiku, Leo Tileni Lucas, Evelyn Nanjeke Mwenda, Simbarashe Chinyoka, Georgina Tjipura-Zaire, Frieda Shilongo, Christian Borgemeister, Siegfried Khaiseb, Simbarashe Chitanga, Sandra Junglen

**Affiliations:** ^1^Department of Preclinical Veterinary Studies, School of Veterinary Medicine, Faculty of Health Sciences and Veterinary Medicine, University of Namibia, Windhoek, Namibia; ^2^Institute of Virology, Charité–Universitätsmedizin Berlin, Corporate Member of Freie Universität Berlinand Humboldt-Universität zu Berlin, Berlin, Germany; ^3^Central Veterinary Laboratory, Directorate of Veterinary Services, Ministry of Agriculture, Water and Land Reform, Windhoek, Namibia; ^4^Center for Development Research (ZEF), University of Bonn, Bonn, Germany

**Keywords:** *Brucella*, Rift Valley fever, seroprevalence, cattle, interface, One Health

## Abstract

**Introduction:**

Brucellosis and Rift Valley fever (RVF) are neglected zoonotic diseases (NZD) that threaten public health, animal health, and production in resource-limited countries including Namibia.

**Methods:**

The objective of this cross-sectional study was to determine *Brucella* spp. and RVFV seroprevalence in cattle at the wildlife-livestock interface in the Kabbe South constituency (Zambezi region) of Namibia. Cattle sera (*n* = 371) were randomly collected from 18 cattle herds in six constituency areas and tested for antibodies against *Brucella* [complement fixation test (CFT) and indirect enzyme-linked immunosorbent (ELISA) assay in parallel] and Rift Valley fever virus (competitive ELISA).

**Results:**

Apparent individual animal prevalence for *Brucella* spp. was 5.9% (95% CI: 3.95%−8.81%, 22/371) and 20.8% (95% CI: 16.9%−25.2%, 77/371) based on the CFT and I-ELISA, respectively. For RVFV, apparent and true animal prevalence were 41.0% (95% CI: 36.1%−46.0%, 152/371) and 47.6% (95% CI: 41.8%−53.6%), respectively. Animal and true prevalence of *Brucella* spp. based on the CFT and ELISA in parallel were 22.6% (95% CI: 18.7%−27.2%, 84/371) and 19.7% (95% CI: 15.6%−24.4%), respectively. About 10.8% (40/371) of cattle tested positive for both *Brucella* spp. and RVFV antibodies. Prevalence of *Brucella*-positive cattle herds was 83.3% (15/18). Within herd *Brucella* spp. seroprevalence was 0%−70%. All cattle herds tested positive for RVFV, with prevalence of 1.7% to 70%. Binomial logistic regression revealed that sex was a significant predictor (*p* < 0.05) for RVFV seropositivity, but not for *Brucella* spp. seropositivity (*p* > 0.05). Test agreement between CFT and I-ELISA when used for the detection of anti-*Brucella* antibodies was poor (k = 0.2322).

**Discussion:**

*Brucella* spp. and RVFV infections were prevalent in communal pastoral cattle at the human-wildlife-livestock interface in the Zambezi region suggesting a higher likelihood of occurrence of reproduction losses in cattle and zoonotic disease in humans. We recommend the enforcement of the requirements for the vaccination of heifers against brucellosis in the affected communal areas to reduce the risk of human infection. The use of One Health principles for the surveillance, prevention and control of *Brucella* spp. and RVFV infections can promote the effective control of these zoonotic infections at the interface.

## 1 Introduction

Livestock production is the main source of livelihood for most rural communities in Africa, including Namibia (1). However, the sector faces challenges from infectious diseases that cause losses in the productive and reproductive performance of livestock, such as brucellosis and Rift Valley fever (RVF). RVF and brucellosis are neglected tropical diseases (NTD) that are frequently under-diagnosed and under-reported in developing countries (2).

Brucellosis is caused by gram negative bacteria of the genus Brucella in humans and a wide variety of domesticated and wild animals (3). Infection of livestock causes abortions, stillbirth, reduced fertility, poor weight gain, and decline in milk production. Animal and animal product trade in affected regions is restricted. The disease is thus of economic importance (4). Bovine brucellosis is primarily caused by *Brucella abortus* and occasionally by *Brucella melitensis* and *Brucella suis* (5). It is transmitted among cattle through contact with or ingestion of food contaminated with aborted material such as fetuses, fetal membranes and vaginal secretions. Human infection is common among professionals who work with cattle and handle their products (meat, raw milk, and dairy products), such as farmers, farm workers, veterinary personnel, and butchers (6). The infection can manifest with mild non-specific clinical symptoms which are clinically indistinguishable from common human febrile diseases, like malaria and influenza.

Bovine brucellosis is endemic in Southern Africa. An epidemiological study conducted in South Africa from 2007 to 2015 revealed an overall seropositivity of 5.85% (44,687/764,276) for brucellosis in livestock (7). Bovine brucellosis prevalence at the wildlife-livestock interface in southern Africa is rarely reported. However, studies in Mozambique, Zimbabwe and Zambia have reported prevalence of 9.77%, 9.9% and 19% respectively (8–10). In Namibia, brucellosis has been reported in cattle, sheep, and goats on the basis of clinical signs and serological surveys (11–15). The seroprevalence of bovine brucellosis in livestock in Namibia ranges from 0.49% to 1.94%, while the prevalence of human brucellosis is estimated to be 11.64% (11, 16). Brucella disease control strategies in Namibia involve vaccination, surveillance and culling of infected animals. However, as bovine brucellosis is endemic in most areas of Southern Africa, the control of the disease is difficult to achieve, especially under conditions of nomadic or migratory husbandry practices where hygienic precautions are difficult to implement.

Rift Valley Fever (RVF) is a zoonotic mosquito-borne disease of sheep and goats that can also infect cattle and antelope species. *Aedes* spp. mosquitoes are the primary vectors of RVF virus (RVFV) in Africa, but *Culex* spp. mosquitoes can also transmit the virus (17). Mosquitoes transmit RVFV via blood-feeding to susceptible livestock and humans. The virus can also be transovarially transmitted to the next generation of *Aedes* mosquitoes (18). In southern Africa, the emergence of transovarially infected *Aedes* spp. mosquitoes causing RVFV outbreaks in animals and humans is linked to persistent heavy rainfall, flooding associated with the El Niño phenomenon and floods occurring at the end of the rainy season (19, 20). RVFV outbreaks are often followed by long inter-epidemic intervals that may last for many years. Climate change is predicted to expand the geographical range of RVFV and shorten the period between outbreaks (21). Human infection may also occur by direct contact with infected blood, organs, fetuses and raw meat, and to a lesser extent by drinking unpasteurized milk. RVFV causes high abortion rates in pregnant animals and typically high mortality in young animals (95%−100%), as well as non-specific, febrile influenza-like infections in humans (5) that can be mistaken for malaria and other febrile diseases. RVFV can also lead to hepatitis, encephalitis and retinitis in humans (22, 23). It is primarily an occupational disease of professionals such as butchers, farmers, farm workers, herders and veterinary personnel. In Africa, seroprevalence of RVFV in cattle and sheep can reach 100% (20). RVFV caused epidemics in livestock in the southern and western parts of Namibia in 2010 and 2011 (24). Despite its public health and socioeconomic impact, no studies investigating seroprevalence rates in cattle in Namibia have been conducted to date.

The tropical climate (hot and humid) and the human-wildlife-livestock interface areas in the Zambezi region present ideal conditions for the survival and transmission of zoonotic pathogens including *Brucella* and RVFV to resource-limited communities and domestic animal species inhabiting these areas. Seasonal flooding that occurs in the eastern Zambezi region is ideal for the proliferation of mosquito vectors of RVFV, which can lead to disease outbreaks.. Previous studies on brucellosis focused on commercial livestock farming systems, where animal disease control measures are better implemented, rather than on communal farming systems, where animal disease controls such as vaccination are not applied and herds share grasslands. Therefore, the objective of this study was to determine the seroprevalence of *Brucella* and RVFV in a rural communal area of the Zambezi region in Namibia and recommend appropriate prevention and control strategies for cattle and humans.

## 2 Materials and methods

### 2.1 Study area

**Figure 1:**
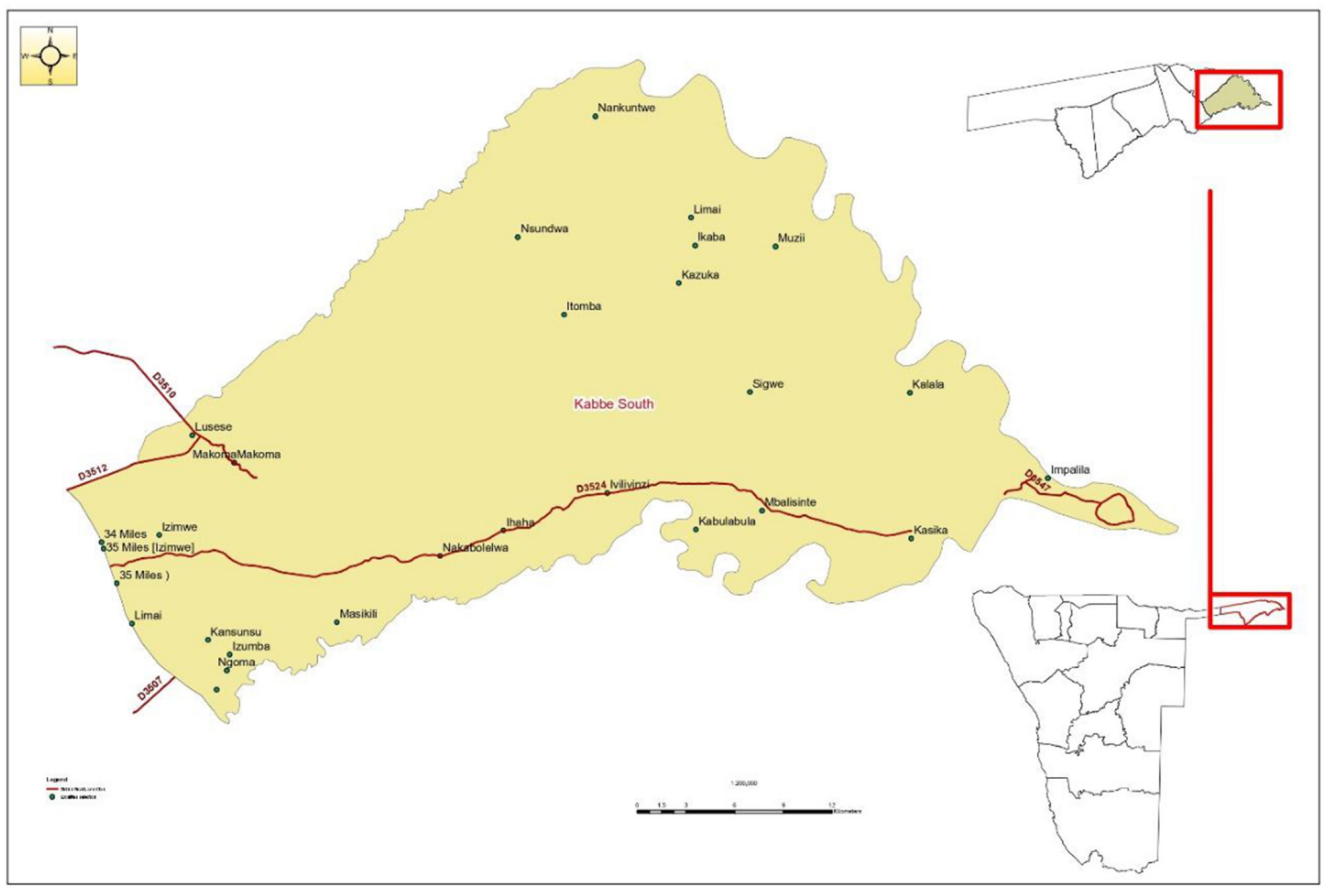
Map of Kabbe South constituency showing the location of the study areas in Zambezi region (Namibia).

The study was carried out in the rural part of Kabbe South Constituency in the Kavango Zambezi Transfrontier Conservation area (KAZA TFCA) in north-eastern Namibia (Figure 1). Kabbe South is located at 17° South, 23° East in the eastern part of the Zambezi Region, about 79 km from the regional capital Katima Mulilo, and is one of the eight constituencies in the region. The constituency is a major cattle farming area with a population of 11,345 inhabitants, 473 of whom are registered cattle farmers. Seasonal flooding and the resultant migration of people and cattle between low and high ground is a feature of this area. Kabbe South shares borders with Zambia, Zimbabwe, and Botswana. In the KAZA TFCA, wild animals roam freely across international borders and frequently come into direct and indirect contact with cattle raised on communal land.

### 2.2 Study population

The study cattle were reared on communal grazing land, where they came into regular direct or indirect contact with wild herbivores such as buffalo, zebra, springbok, elephants, waterbuck, and hippopotamus. Cattle herds recruited for the study were those that were raised within the wildlife-livestock interface area and had not been vaccinated against *Brucella* and RVFV infections.

### 2.3 Study design

As a cross-sectional pilot survey, the aim of our study was to determine the seroprevalence of *Brucella* spp. and RVFV in cattle at a wildlife-livestock interface in Kabbe South constituency of the Zambezi region. Kabbe South constituency was purposely selected to target an area where cattle and wild herbivores mingle. Within the constituency, six out of 24 areas (Sigwe, Kasika, Nantungu, Kabulabula, Muzii, and Ivilivinzi) were selected for the study using the simple random sampling technique. Systematic random sampling was used to choose 18 cattle herds and 371 cattle of various ages, breed, and sex, for blood sampling at crush pens in 20 villages. Bovines greater than 6 months of age were considered for the study.

### 2.4 Sample size

The formula *n* = 4PQ/L2 by Martin et al. (25), where P is the expected prevalence, Q = 1-P and L is the desired precision, was used to calculate the sample size for estimating seroprevalence. In this calculation, we assumed a 5% seroprevalence of *Brucella* spp. and RVFV prevalence in the cattle population, a precision of 0.025 (2.5%) and a 95% level of confidence to calculate a sample size of 304. However, 371 samples were collected to account for possible effects of clustering.

### 2.5 Blood sampling and serum recovery

From November to December 2023, blood (5 ml, *n* = 371) was collected aseptically from the jugular vein of randomly selected rope or crush pen-restrained cattle into sterile plain vacutainer tubes. For each sample, information regarding the date of sampling, animal identification, age, sex, breed, location, vaccination status, and contact with wild ruminants was recorded. The blood samples were transported on ice to the regional laboratory at Katima Mulilo. After overnight storage, sera were recovered by centrifugation at 3,000 rpm for 5 min. They were identified, securely packed on ice and dispatched to the Central Veterinary Laboratory (CVL) (Windhoek) where they were kept frozen at −20°C until tested for *Brucella* spp. and RVFV infection.

### 2.6 *Brucella* and RVFV serological analysis

Serological tests for anti-*Brucella* spp. and anti-RVFV antibodies were carried out at the Central Veterinary Laboratory (CVL) (Windhoek, Namibia). The standard tests for the diagnosis of *Brucella* spp. infection at CVL are the Rose Bengal plate test (RBPT) and the compliment fixation test (CFT) in series. However, in this study, the *Brucella* complement fixation test (CFT) and the indirect enzyme-linked immunosorbent assay (I-ELISA) were used in parallel to detect anti-*Brucella* spp. antibodies and to compare the performance of the two assays.

The CFT was performed following the procedure described by the World Organization for Animal Health (26), using standardized antigens (*B. abortus* Weybridge strain 99) that detect O smooth anti-*Brucella* antibodies stimulated by smooth *Brucella* spp. Results for the CFT were read after the plates had been left to stand for 1 h to permit unlysed cells to settle. CFT test results of 30ICFTU/ml and above were considered positive based on the absence of hemolysis.

The enzyme-linked immunosorbent assays (ELISAs) used to detect anti-*Brucella* and anti-RVFV IgG antibodies in serum were the indirect ELISA (I-ELISA) and the competitive ELISA (C-ELISA), respectively. These were performed in duplicate using commercial ELISA test kits, the ID Screen^®^ Brucellosis Serum Indirect Multi-species and ID Screen^®^ Rift Valley Fever Competition Multi-species (IDvet, Innovative Diagnostics, Grabels, France, https://www.innovative-diagnostics.com). The ELISA assays were performed and validated following the manufacturers' procedures and cut-off points for distinguishing positive and negative results, without any modifications. Before analysis, all sera were inactivated at 56°C for 30 min. Test results were read as positive or negative. Positive and negative controls were included in each test run for test validation.

### 2.7 Data analysis

Data analysis was performed in SPSS version 24. An animal was considered seropositive if it tested positive on the CFT, I-ELISA or C-ELISA. Cattle herds were considered positive for brucellosis or RVF if at least one animal was seropositive. Apparent seroprevalence was calculated per infection, age, sex, and breed of cattle as the proportion of tested cattle that were seropositive. True prevalence was estimated by adjusting apparent prevalence for test sensitivity and specificity according to Epitools (https://epitools.ausvet.com.au/). The agreement between CFT and I-ELISA assays was tested using Cohen's Kappa analysis. Kappa coefficients >0.75, between 0.4 and 0.75, and < 0.4 were considered as excellent, fair, and poor agreement, respectively. The Chi-square test was used to test for the significance of differences in prevalence between age, sex, and breed, with *p* < 0.05 as the significance level. A binomial logistic regression model was applied to assess the relationship between age, sex and breed, and testing positive or negative for *Brucella* or RVFV infection, as determined by the *Brucella* CFT and I-ELISA (in parallel) and the RVFV C-ELISA assays.

### 2.8 Ethics statement

The University of Namibia Research Ethics Committee (Ex2/6/23) and the Directorate of Veterinary Services (Ministry of Agriculture, Water and Land Reform) approved the study protocol. Informed consent was sought from cattle owners before blood sampling. Blood collection was performed humanely in crush pen or rope-restrained cattle.

## 3 Results

A total of 371 serum samples from cattle were collected from 18 herds in the communal areas of Kabbe South Constituency. The majority of these samples were from females (80.1%, 297/371) and cattle of breeding age, i.e., ≥2 years (95.7%, 355/371). Samples were drawn from the following cattle breeds: Sanga (80.6%, *n* = 299), Brahman (12.1%, *n* = 45), Simbra (0.5%, *n* = 2), Limousine (0.3%, *n* = 1), Swiss (0.3%, *n* = 1), and crossbreeds (6.2%, *n* = 23). Most cattle (69%, 256/371) were 4 years old.

### 3.1 Apparent and true seroprevalence of *Brucella* spp.

The apparent seroprevalence of *Brucella* spp. infection in cattle was 5.9% (95% CI: 3.95%−8.81%, 22/371) and 20.8% (95% CI: 16.9%−25.2%, 77/371) based on the CFT and I-ELISA assays, respectively (Table 1). The overall prevalence of *Brucella* spp. infection based on the combined number of reactors detected by both assays in parallel (matching results were counted as one) was 22.6% (95% CI: 18.7%−27.2%, 84/371).

**Table 1:** Apparent and true seroprevalence of *Brucella* spp. and RVFV infections in the Kabbe South constituency.

**Serological test**	**Number of sera tested**	**Number positive**	**Apparent prevalence (%) (95% CI)**	**True prevalence (%) (95% CI)**
*Brucella* I-ELISA	371	77	20.8 (16.94%−25.17%)	19.7 (15.6%−24.4%)^*^
*Brucella* CFT	371	22	5.9 (3.95%−8.81%)	
Combined *Brucella* CFT and I-ELISA (parallel)	371	84	22.6 (18.68%−27.17%)	
RVF C-ELISA	371	152	41.0 (36.08%−46.04%)	47.6 (41.8%−53.6%)

^*^Calculated using combined sensitivity and specificity of CFT and I-ELISA assays used in parallel; CI, confidence intervals.

The true prevalence of *Brucella* spp. was estimated by computing the combined sensitivity (Se) and specificity (Sp) of the CFT and I-ELISA using Epitools (https://epitools.ausvet.com.au/). The resultant Se and Sp, and the overall number of positive cases resulting from using the two tests in parallel, were used to estimate true prevalence (Table 1). *Brucella* CFT Se and Sp of 95% and 100% respectively (27), and I-ELISA Se and Sp of 96.8% and 96.3% in cattle (28) respectively, were used for the calculation. The combined Se and Sp was 99.84% and 96.3% respectively. Therefore, the estimated true prevalence of *Brucella* spp. infection was 19.7% (95% CI: 15.6%−24.4%).

### 3.2 Apparent and true seroprevalence of RVFV

For RVFV, the apparent individual animal prevalence was 41.0% (95% CI: 36.1%−46.0%, 152/371) (Table 1). True prevalence for RVFV was estimated at 47.6% (95% CI: 41.8%−53.6%) based on C-ELISA test sensitivity and specificity of 85.4% and 98.6%, respectively (27).

Cattle (*n* = 40, 10.8%) consisting mainly of cows (82.5%, 33/40), tested positive for both *Brucella* spp. and RVFV antibodies on the assays used.

### 3.3 Herd seroprevalence of *Brucella* spp. and RVFV

The prevalence of *Brucella*-positive cattle herds based on the CFT and I-ELISA assays was 66.7% (12/18) and 83.3% (15/18) respectively. All cattle herds that tested positive on the CFT were also positive on the I-ELISA, giving an agreement rate of 80% (12/15) between the results of the two assays. The overall herd prevalence of *Brucella* spp. infection based on the combined results of the CFT and I-ELISA (83.3%, 95% CI: 60.8%−94.2%) was the same as the herd prevalence determined using the I-ELISA assay alone. Within the tested herds, *Brucella* spp. seroprevalence ranged from 0%−18.2% (CFT) and 0%−70% (I-ELISA).

The prevalence of RVFV positive cattle herds was 100% (*n* = 18). Within-farm prevalence varied from 1.7%−70%.

### 3.4 Seroprevalence of *Brucella* spp. by age, sex, and breed

The prevalence of *Brucella* positive sera (7.0%, 18/256, CFT; 23.0%, 59/256, I-ELISA) was high in females, cattle of 4 years and older, and in exotic breeds, but this was not significant (Table 2). Bovine *Brucella* spp. seroprevalence (based on CFT and I-ELISA in parallel) was higher in females (23.9%. 71/297) than males (17.6%, 13/74), but the differences were not significant (*p* = 0.28). Most positive sera for *Brucella* (*n* = 59) were from the Sanga breed of cattle.

**Table 2:** Binomial logistic regression assessment of the association between age, sex, and breed, and the presence or absence of bovine *Brucella* spp. antibodies as determined by the CFT and I-ELISA in parallel.

**Predictor**	**Tested animals**	**Number positive**	**% positive**	**Estimate**	**SE**	**Z**	**p**	**Odds ratio (95% CI)**
**Intercept**				−2.006	0.837	−2.397	0.017^*^	0.135 (0.0261–0.694)
**Age**								
≤ 2	24	2	8.3	-	-	-	-	-
>2– < 4	88	15	17.0	0.796	0.847	0.939	0.348	2.216 (0.4212–11.567)
≥4	259	67	25.9	1.163	0.768	1.513	0.130	3.200 (0.7095–14.429)
**Sex**								
Male	74	13	17.6	-	-	-	-	-
Female	297	71	23.9	0.659	0.363	0.990	0.322	1.432 (0.7034–2.915)
**Breed**								
Crossbreeds	25	8	32.0	-	-	-	-	-
Brahman	45	11	24.4	−0.400	0.561	−0.714	0.475	0.670 (0.2233–2.011)
Exotic breeds	2	1	50.0	0.663	1.489	0.446	0.656	1.942 (0.1048–35.953)
Sanga	299	64	21.4	−0.682	0.464	−1.469	0.142	0.506 (0.2035–1.256)

^*^Significant at *p* < 0.05.

#### 3.4.1 Binomial regression model assessment of the effect of age, sex, and breed on *Brucella* spp. seropositivity

A binomial logistic regression model was performed to ascertain the relationship between age, sex and breed, and seropositivity for *Brucella* spp. as determined by a combination of CFT and I-ELISA carried out in parallel. The model displayed a relatively low pseudo-R^2^ (McFadden's R^2^ = 0.0205), indicating that the predictors explained approximately 2.05% of the variation in the outcome variable (seropositive or seronegative for *Brucella* spp.). The intercept of the model was statistically significant (Estimate = −2.006, SE = 0.837, *p* = 0.017), indicating that the log odds of disease presence were significantly different from zero. The model had an overall accuracy of 72.2%, which means that the model correctly predicted *Brucella* seropositive or seronegative cases 72.2% of the time. This performance was largely driven by the model's high specificity (89.2%). Its sensitivity was low (14.3%). The area under the curve (AUC) for the receiver operating characteristic (ROC) was 0.577, indicating moderate discrimination between positive and negative *Brucella* spp. cases (above random chance AUC of 0.5).

Results of the logistic regression are depicted in Table 2. Several categories within the age, breed, and sex variables showed odds ratios that were not statistically significant. Thus, age, sex, and breed were not significant predictors of *Brucella* spp. seropositivity (*p* > 0.05).

### 3.5 Seroprevalence of RVFV by age, sex, and breed

For RVFV, seroprevalence was higher in females (44.8%, 133/297) than male (25.7%, 19/74) cattle (*p* = 0.002) (Table 3). RVFV seroprevalence was detected in all age groups of cattle including young animals (< 2 years old). Most positive sera for RVFV (*n* = 121) were from the Sanga breed of cattle (Table 3). These sera represented 79.6% (121/152) of the total positive sera recorded for RVFV in this study.

**Table 3:** Binomial logistic regression assessment of the relationship between age, sex, and breed, and the presence or absence of RVFV antibodies as determined by the C-ELISA assay.

**Predictor**	**Tested animals**	**Number positive**	**% positive**	**Estimate**	**SE**	**Z**	***p***	**Odds ratio (95% CI)**
**Intercept**				−0.6499	0.584	−1.112	0.266	0.522 (0.166–1.64)
**Age**								
≤ 2	24	9	37.5	-	-	-	-	-
>2– < 4	88	21	23.9	−1.1172	0.582	−1.920	0.055	0.327 (0.105–1.020)
≥4	259	122	47.1	−0.1752	0.473	−0.3705	0.711	0.839 (0.322–2.12)
**Sex**								
Male	74	19	25.7	-	-	-	-	-
Female	297	133	44.8	0.9960	0.326	3.0579	0.002^*^	2.708 (1.43–5.13)
**Breed**								
Crossbreeds	25	11	44.0	-	-	-	-	-
Brahman	45	18	40.0	−0.0772	0.526	−0.1467	0.883	0.926 (0.330–2.60)
Exotic breeds	2	2	100	14.9317	599.538	0.0249	0.980	3.05 × 10^6^ (0–∞)
Sanga	299	121	40.5	−0.3352	0.441	−0.7608	0.447	0.715 (0.302–1.70)

^*^Significant at *p* < 0.05; ∞, infinity.

#### 3.5.1 Binomial regression model assessment of the relationship between age, sex, and breed on RVFV seropositivity

A logistic regression model was used to assess the relationship between predictors (age, sex, and breed) and seropositivity of RVFV as determined by the C-ELISA assay. The model displayed a relatively low pseudo-R^2^ (McFadden's R^2^ = 0.0439) (Table 3), indicating that the predictors explained approximately 4.0% of the variation in the outcome variable. The intercept of the model was not statistically significant (Estimate = −0.6499, SE = 0.584, *p* = 0.266), indicating that, in the absence of the predictors, the log odds of disease presence (C-ELISA) are not significantly different from zero. This suggests no inherent bias toward disease presence or absence without considering other predictors. The model had an overall accuracy of 59.6%. It correctly predicted RVFV seropositive or seronegative cases about 60% of the time. It showed a relatively high specificity (90.0%), while its sensitivity was low (15.8%).

Sex was a significant predictor of seropositivity (Table 3). The odds of RVFV seropositivity were 2.708 times higher in females than in males (Odds Ratio = 2.708, 95% CI: 1.430 to 5.13, *p* = 0.002), with females being at a higher risk of RVFV. The age group 2.5 years had a significant negative association with RVFV seropositivity (Estimate = −1.98749, SE = 0.979, *p* = 0.042). All age categories, such as >2– < 4 years (Odds Ratio = 0.327, *p* = 0.055) and ≥4 years (Odds Ratio = 0.839, *p* = 0.711), did not show statistically significant associations, indicating that their odds of seropositivity were not markedly different from the reference group (≤ 2 years).

The area under the curve (AUC) for the receiver operating characteristic (ROC) was 0.609, indicating moderate discrimination between seropositive and seronegative cases as it is above random chance (AUC = 0.5).

### 3.6 Comparison of CFT and I-ELISA performance in detecting antibodies against *Brucella* spp.

The performance of the *Brucella* CFT and I-ELISA assays on test samples was compared using the Cohen's Kappa test of agreement (Table 4). Test agreement between CFT and I-ELISA when used for the detection of *Brucella* infection (antibodies) was poor (k = 0.2322) (Table 4). In cattle aged ≤ 2.5 years, the CFT did not detect any reactive *Brucella* sera, whereas the I-ELISA identified six of these animals as seropositive.

**Table 4:** Kappa test of agreement between *Brucella* CFT and I-ELISA.

	***Brucella*** **I-ELISA**		Kappa value^*^	**Interpretation**	***P*-value**
**CFT**	**Positive**	**Negative**	0.2322	Poor agreement	0.001^*^
Positive	15	7			
Negative	62	287			

^*^Significant at *p* < 0.05; Kappa values: >0.75 = excellent agreement, 0.4–0.75 = fair agreement, and < 0.4 = poor agreement.

## 4 Discussion

Brucellosis and Rift Valley fever are important zoonotic diseases in southern Africa, affecting human and livestock health and can have a great economic impact in regions where livelihoods depend on livestock farming. Their epidemiology is poorly understood, especially in rural regions at the wildlife-livestock interface where diagnostic resources are limited. In this study, we aimed to determine the exposure of cattle to *Brucella* spp. and RVFV in Northern Namibia. We found a relatively high seroprevalence of *Brucella* spp. and RVFV in apparently healthy communal pastoral cattle. To our knowledge, this is the first report of RVFV seroprevalence in cattle in Namibia.

Our study confirms that brucellosis is endemic in cattle managed under a communal pastoral system at the human-wildlife-livestock interface, with a relatively high individual animal and herd seroprevalence of 22.6% and 83.3%, respectively. The individual animal prevalence was higher than the approximately up to 2% in cattle drawn mainly from commercial farms reported previously in the southern drier part of Namibia (11, 14, 15), where preventive and control measures for brucellosis are strictly implemented. The elevated prevalence found in this study is consistent with a higher brucellosis prevalence of 10.3% recorded previously in communal pastoral cattle in Namibia (15). A relatively high prevalence of *Brucella* reactors (15.6%) has also been reported in communal cattle in the Kwa-Zulu Natal province of South Africa (29) and from several surveys in communal pastoral cattle in Africa (30–32). In contrast, findings from other neighboring countries reported lower seroprevalence rates of 8% in cattle from Zambia (33), 6% in cattle from South Africa (7) and 6% in buffalo from Botswana (34). The high individual animal and herd prevalence in this study point to a relatively increased level of zoonotic disease burden in cattle and risk to human health, as the prevalence of positive reactors within herds was as much as 70%. Moreover, elevated *Brucella* seroprevalence values in livestock, as recorded in our study, have been linked to seropositivity in the human population (35). The high prevalence of *Brucella* reactors in communal areas can be ascribed to a communal pastoral system of cattle management that promotes intermingling between cattle herds; the sharing of pastures, water, and breeding bulls; and a lack of awareness of the disease, all of which promote disease transmission. Direct and indirect contact between cattle herds and between cattle and wild ruminants at the wildlife-livestock interface brings infected aborting and birthing animals into close contact, increasing the risk of *Brucella* transmission (36). Identified risk factors for brucellosis persistence and failure of eradication in developing countries comprise inefficient surveillance and control programs that become evident in the inappropriate disposal of aborted materials, the lack of knowledge and awareness in medical professionals, veterinarians, and in traditional livestock owners, reluctance of vaccination, uncontrolled animal movement and lack of differential diagnostics that include biotyping methods (37). The fact that our sample consisted mainly of cows kept in herds for longer periods may have contributed to higher exposure and prevalence of *Brucella* infection (38). In addition, contact with potential wild ruminant reservoirs of *Brucella abortus* such as buffalo (39) and wildebeest (40) in this interface area could have contributed to the high proportion of *Brucella* reactors seen in cattle. Our results point to the possibility of high reproduction losses due to infertility, abortions, and neo-natal mortalities associated with *Brucella* and RVFV infections in communal cattle and a high risk for zoonoses in the human population. The I-ELISA assay used in this study detects IgG anti-*Brucella* antibodies. Therefore, we can conclude that positive reactors were animals that carried chronic infections or that had recovered from previous infection.

We further found a relatively high individual animal (41%) and herd seroprevalence (100%) for RVFV, in line with previous reports of RVF being an endemic disease in Africa with a prevalence of up to 100% (20). The overall prevalence of RVFV in cattle was similar to a prevalence of 42.9% in a vaccinated population in South Africa (41), but higher than the 12.37%−33.3% reported in other African countries (42–46). Notably, the RVFV prevalence in cattle in the current study was comparable to that reported previously in springbok (*Antidorcas marsupialis*) (35%) and black-faced impala (*Aepyceros melampus petersi*) (62.5%) in a national park in Namibia (47), perhaps a reflection of the potential role of wildlife in the transmission cycle. Wild animals in the study area, such as buffalo, waterbuck, elephant, eland, and wildebeest, may have acted as maintenance hosts for RVFV, from which spillover infection was transmitted to domestic animals through infected mosquitoes (48). With a 100% herd prevalence, our results suggest a high burden and circulation of RVFV in cattle at the wildlife-livestock interface in the Kabbe South constituency. These findings reveal an animal and public health risk, as seroprevalence rates in domestic animals correlate positively with the risk of infection in humans (49). Interestingly, no RVFV outbreaks have ever been reported in this part of the country, despite the presence of established animal disease surveillance structures; occurrence of favorable ecological and climatic conditions for the breeding of *Aedes* or *Culex* mosquito vectors in the form of frequent episodes of heavy rainfall, seasonally flooded areas, wetlands/dambos, and permanent water bodies (50). The detection of seropositive cattle suggests the current circulation of the virus or the long-term persistence of anti-RVFV antibodies due to previous infections. Of note, the cattle of this study were not vaccinated against RVFV. The silent circulation of RVFV in animals has also been documented by previous studies (51–53). Cattle are more attractive to RVFV mosquito vectors than other livestock species (54), hence an increased prevalence is often seen in these species. The high RVFV seroprevalence observed in cattle could also be due to spillover of infection from infected wild animals that frequently mingle with and share grazing and water resources with cattle. Previous studies in Namibia have provided serological evidence of the role of wildlife such as springbok (*Antidorcas marsupialis*), wildebeest (*Connochaetes taurinus*), and black-faced impala (*Aepyceros melampus petersi*) in the epidemiology of RVFV (47). Moreover, the study area falls within the Kavango-Zambezi Transfrontier Conservation Area (KAZA-TCFA), an area where wild animals such as buffalo and wildebeest roam freely across five international borders (Botswana, Zambia, Zimbabwe, Namibia, and Angola).

It is noteworthy that several cattle (10.8%, *n* = 40) were seropositive for both *Brucella* and RVFV, suggesting successive or co-exposure to the two pathogens. Either way, our findings point to significant reproductive challenges in the affected herds and a public health risk in the respective communities.

In this study, established risk factors for *Brucella* infection, that is, age, sex and breed (55), were not significantly associated with seropositivity, although seropositivity was higher in females than males, and in sexually mature (4 years and older) than younger animals. Our findings are consistent with previous reports which noted a higher *Brucella* seroprevalence among sexually mature cattle (36, 56) and in cows (57, 58). In contrast to the findings in Malawi (46) and Madagascar (42), our study established a higher RVFV seroprevalence in females than males (*p* = 0.004), perhaps due to the larger number of female cattle that were recruited in the study. Male cattle are often culled, resulting in reduced exposure time, while cows stay longer and dominate the herds (58). RVFV seropositivity occurred across all age groups of cattle tested, with higher but not significant prevalence rates in adults than in younger animals, in agreement with prior studies (46). As expected, the Sanga breed, the major cattle breed in the study area, had a higher seroprevalence of *Brucella* and RVFV than other breeds.

Test agreement between *Brucella* CFT and I-ELISA was poor (kappa value of 0.2322). Previous studies have confirmed that I-ELISA has a higher sensitivity and specificity than CFT (27), which explains our results. In our study, I-ELISA detected *Brucella* antibodies in younger cattle (≤ 2 years), while the CFT did not, and all cattle herds that tested positive on the CFT, also tested positive on the I-ELISA, confirming its superiority over the CFT. We therefore recommend that the Central Veterinary Laboratory (Namibia) adopt the I-ELISA as a confirmatory test (after RBPT screening) in place of the CFT.

Our findings highlight the need to prioritize zoonoses in the country and to establish a One Health policy for effective surveillance, prevention and control of zoonotic pathogens such as *Brucella* and RVFV, as has been implemented elsewhere in Africa (59–61). This is because *Brucella* and RVFV infections have multidimensional determinants of disease epidemiology that include human, domestic animal, wildlife, climatic, and ecological factors. The One Health approach should foster collaboration between professionals working in different sectors such as human health, animal health and production, entomology, social sciences, wildlife, and the environment (62). In particular, the public health and animal health sectors need to establish regular communication, collaboration and information sharing in the surveillance, prevention, and control of zoonotic infections. We recommend that the public health sector increase awareness of these infections among health professionals to ensure effective surveillance and detection in high-risk populations at the interface. Vaccination of cattle against brucellosis has been shown to reduce infection, transmission and disease burden. It should therefore be implemented in communal areas. Further studies on mosquito vectors, humans, and domestic and wild ruminants are recommended, using serological, molecular, culture, and virus isolation techniques to characterize circulating strains of *Brucella* and RVFV.

The inability to identify *Brucella* spp. infecting animals in Namibia may reduce the effectiveness of control measures that are currently implemented in cattle and humans, as all smooth *Brucella* spp. can cause infection singly or in co-infections. Therefore, the characterization of *Brucella* spp. infecting cattle should be the focus of future studies.

### 4.1 Limitations

Some cattle herds in flooded and muddy areas could not be reached due to bad or non-existent roads. The *Brucella* CFT and I-ELISA assays used in this study cannot distinguish between smooth field *Brucella* species. Positive results serve only to confirm that the animals were exposed to *Brucella* spp. Moreover, serological tests may fail to detect *Brucella* antibodies during the early stages of the infection (up to 12–16 days) or cross-react with other gram negative bacteria, which may underestimate or overestimate seroprevalence respectively. The performance of the CFT can be limited by hemolyzed sera or sera with anti-complementary activity. The CFT is a very complicated assay, that requires specialized facilities, equipment, time and personnel, which can interfere with its performance and results. However, in this study, the drawbacks of the CFT were compensated for by the simpler and highly sensitive I-ELISA assay that was used in parallel.

## 5 Conclusion

Our study determined a relatively high seroprevalence of *Brucella* spp. and RVFV antibodies in cattle raised in a communal pastoral system at the wildlife-livestock interface in Northern Namibia. Sex was a significant risk factor for RVFV, with females having a higher likelihood of infection. In contrast, age, sex and breed were not significant predictors of *Brucella* spp seropositivity. The detection of these zoonotic infections has serious animal and public health implications.

## Data Availability

The original contributions presented in the study are included in the article/supplementary material, further inquiries can be directed to the corresponding author.
